# The unintended consequences of tolerance: The experience and repercussions of being tolerated for minority group members

**DOI:** 10.1371/journal.pone.0282073

**Published:** 2023-03-22

**Authors:** Levi Adelman, Kumar Yogeeswaran, Maykel Verkuyten

**Affiliations:** 1 ERCOMER, Utrecht University, Utrecht, Netherlands; 2 School of Psychology, Speech and Hearing, University of Canterbury, Christchurch, New Zealand; Universidad Central de Chile, CHILE

## Abstract

Tolerance as forbearance implies the decision to not interfere when others engage in actions or beliefs that you disapprove of. This allows minorities to live the life that they want, despite the disapproval. However, the undercurrent of disapproval which comes with tolerance might carry unintended negative consequences for tolerated minorities. The present research utilizes a novel experimental method to give participants an experience of being tolerated to address two key questions: 1) what are the consequences of being tolerated on personal well-being? 2) how does the experience of being tolerated affect future expectations and willingness to raise one’s voice? Across four studies with American and Dutch participants (*N* = 1,758), we find that being tolerated leads to less positive outcomes on well-being than being accepted (although more positive implications relative to being rejected). Further, being tolerated reduces the expectation of being valued in future encounters while simultaneously tamping down on people’s willingness to raise their voice against those who begrudgingly include them.

## Introduction

“Tolerance is like a gift that you don’t really want. Many people see tolerance as a positive thing, but is it really? …. On the one hand, tolerance can prevent oppression, but on the other it means that you see the other as inferior or undesirable” [1]

Forbearance tolerance involves enduring and permitting what we disapprove of or find objectionable and, thus, not interfering with or restricting the lives of those who hold contrasting or contradictory beliefs [2–4]. Tolerance is therefore considered a central feature of democratic culture and protects the expression of minority identities and enables diversity [3–5]. However as indicated in the quote above, tolerance might also possess a more sinister side which signals to minorities that their beliefs, practices, and values are undesirable, and merely put up with. A theoretical process model of being tolerated has been proposed that focuses on the asymmetrical nature of being tolerated as a minority member and the implied devaluation of minority identity practices and beliefs [6]. This model argues that the experience of being tolerated as begrudging inclusion can have a negative impact not only on immediate minority well-being and expectations about future interactions, but also on their willingness to raise their voice in dissatisfaction to improve their place in organizations, institutions or society more generally [6]. Examining these possible negative personal, interpersonal and societal implications is important for a balanced understanding of the pros and cons of the widely emphasized focus on forbearance tolerance as an indispensable ingredient for managing diversity in organizations, institutions, and society more generally [3].

Recent research involving the perspective of racial and ethnic minorities, sexual minorities, and people with disabilities suggest that the experience of being tolerated falls in between that of full acceptance and complete rejection [7–9]. However, this research relied primarily on correlational designs and people’s own definitions of the experience of tolerance, making it hard to draw firm conclusions of how forbearance tolerance is experienced. Furthermore, previous research has not investigated the theoretically proposed negative interpersonal and voice-related consequences of being tolerated, which are critical to understanding the social implications of tolerance as an approach to managing diversity. The present experimental research therefore aims to fill in these gaps, while also presenting a novel adaptation of the well-known Cyberball paradigm [10] to simulate the experience of being tolerated in order to examine the theoretically proposed psychological and social implications with experimental controls [6]. Across four studies in two countries, we manipulate minority experiences of being tolerated, in comparison to the experiences of being fully accepted and being rejected, in order to examine the effects of toleration on minority well-being [11, 12], on future expectations from interactions with majority members [13], and on minorities’ willingness to raise their voice and express discontent at their devaluation [14, 15]. Thus, collectively, this investigation experimentally establishes the psychological, interactive, and social consequences of being tolerated for minority groups.

### The psychology of being tolerated

Acceptance, rejection, and tolerance each comprise of two components: the initial attitude and the corresponding behavior. In the first two cases, there is no gap between the attitude and the behavior. Acceptance is when you are welcoming in attitude and inclusive in behavior, such as someone who values and welcomes diversity. Rejection is when you are attitudinally negative towards certain beliefs and practices and are exclusive in behavior, such as someone who disapproves of smoking and excludes smokers in their public and private life. By contrast, tolerance sits in between these with the tolerating attitude involving the negativity of rejection that is combined with the behavioral inclusion of acceptance [3], such as someone who disapproves of smoking, but nonetheless includes smokers in their personal and professional circles. Tolerance can, therefore, be characterized as being “intermediate between wholehearted acceptance and unrestrained opposition” [16]. Thus, the psychological implications of being tolerated can be expected to be in-between acceptance and rejection because tolerance shares with acceptance the behavioral inclusion, but differs in underlying attitude, and it shares with rejection the underlying negative attitude, but differs in behavior.

Theoretically, the notion of forbearance tolerance has been analyzed and criticized as patronizing and condescending to those who are being tolerated, which is argued to have negative personal, interpersonal, and voice-raising consequences [6, 17]. Simply knowing that as a minority member one is being tolerated could be offensive and hurtful because it implies the disapproval and devaluation of what minorities believe and practice by the majority around them, and people typically do not find it desirable to be begrudgingly included, but rather want to be valued and respected [18, 19]. Moreover, tolerance may also function as a subtle social mechanism of ‘depoliticization,’ preventing those who are tolerated from feeling morally or practically comfortable with pushing for full acceptance [6, 17, 20]. Therefore, although tolerance protects against oppression and rejection, it might come with the subjective awareness that one is being endured and ‘put up with’ which is expected to negatively affect both minorities’ well-being, and their expectations of how future interactions with majority members might go. Simultaneously, the social message that being tolerant is morally good and praiseworthy might also undermine tolerated minority group members’ willingness to raise their voice and indicate discontent and strive towards a society, community or organization that is more fully accepting of them.

To test these theoretically expected consequences of being tolerated [6] in a systematic way, the current research experimentally simulates the experience of being tolerated (compared to both being accepted and rejected) to test the implications of tolerance for minority well-being, future expectations, and raising voice. While some recent research has explored the implications of being tolerated among people with disabilities or racial, ethnic, and sexual minorities [7–9], such work has largely been correlational or relied on recalled experiences of racial, ethnic, or sexual minority group members, thus capturing a wide range of experiences that people might describe as tolerance without being able to distinguish between them. This inhibits our ability to examine the unique experience of being tolerated and evaluate it separately from many other factors that might influence people’s experiences of tolerance. In the current research, we place participants into a minority position and experimentally manipulate the experience of being begrudgingly included (as opposed to accepted or rejected) to examine its psychological implications for well-being, future engagement, and the willingness to speak out and try to improve the situation.

### Current research

Across four experiments in two countries (USA and Netherlands), we utilize an adaptation of the well-established cyberball paradigm [10, 21] in which a participant plays a ball-toss game with three (virtual) others who allegedly have a different personality work-style. During the game, the participant is ignored and left out or rather equally included in the game. We adapted this paradigm to simulate the experience of being a minority group member interacting with majority group member virtual teammates who express intergroup acceptance (“we welcome diversity”), intergroup toleration (“we will put up with people like you”), or intergroup rejection (“we do not want to work with people like you”). In order to control for past experiences, we randomly placed people into the position of being a minority who would then be accepted, rejected, or tolerated by majority teammates. We opted to test the impact of being tolerated in the context of a study of online work environments which are increasingly introduced in many institutions, companies and organizations, and in which rejection can occur and acceptance is feasible and common, thus offering a reasonable cover story for participants’ experiences. In doing so, we are able to manipulate minority experiences of being tolerated vs. accepted vs. rejected and test its effects on the three key dependent outcomes for minority group members: personal subjective wellbeing, which captures the feelings experienced by a target of tolerance; interpersonal motivation for future participation and engagement with members of the majority, which captures the key criticism that being merely tolerated might lead minority group members self-exclude from interactions with the majority; and willingness to raise one’s voice through offering participants the opportunity to complain or take action against the majority group members, which captures the criticism that tolerance might lead to depoliticization and an unwillingness to push for full acceptance.

We predicted, first, that being begrudgingly included would have a more negative effect on well-being compared to acceptance, but a less negative effect compared to being rejected (H1). Second, we were interested in people’s expectations of future treatment and expected that being tolerated (compared to being accepted) would similarly inhibit expectations of being a valued and included member of the group (but less so compared to being rejected)(H2). Third, we predicted that we would find evidence of depoliticization: that despite experiencing negative consequences for their well-being and future expectations with their teammates, (compared to being accepted), those experiencing tolerance would be less willing to raise their voice to indicate discontent at their devaluation (H3). Thus, while we expect personal well-being and future expectations of positive treatment by the majority to be decreased by being tolerated compared to being accepted, we expect that this will not be reflected in people’s willingness to complain or take action over the treatment, demonstrating a depoliticization effect.

### Method: Overall design

We present four experimental studies with 1,758 total participants (see Table 1), responding to the increasingly recognized importance of conceptual replication [21]. To accomplish this, we examine the impact of being tolerated by using convenient as well as national samples, we include data from two countries with different languages, and we use somewhat different operationalizations of the key outcome variables. This allows us to assess whether the findings are robust across samples, countries, and specific operationalizations. Data collection was conducted in these specific countries for reasons of convenience and because the research team had sufficient cultural knowledge of the national context. Ethical approval for these studies was obtained by the first and last authors at Utrecht University before data collection, and all participants offered online consent to participate in the research. All measures, manipulations, and exclusions in the different studies are disclosed.

**Table 1:** Sample and demographic information for studies 1–5.

Sample	Country	Sampling	Total	Excluded	Analyzed				
Study 1	United States	Cloud/MTurk	412	11	401				
Study 2	United States	Cloud/MTurk	364	4	360				
Study 3	Netherlands	National	610	52	558				
Study 4	United States	Cloud/MTurk	450	12	438				
**Gender**	Female	Male	Other	Refused					
Study 1	47.5%	51.7%	0.7%	0.0%					
Study 2	54.7%	44.7%	0.0%	0.6%					
Study 3	43.5%	56.1%	0.0%	0.4%					
Study 4	47.9%	51.4%	0.7%	0.0%					
**Age**	18–25	26–35	36–45	46–55	56–65	66–74	75+		
Study 1	9.5%	37.8%	27.9%	15.7%	5.7%	3.2%	0.2%		
Study 2	8.1%	33.6%	28.9%	16.4%	9.7%	3.1%	0.3%		
Study 3	10.8%	17.0%	18.8%	15.2%	16.5%	17.4%	4.3%		
Study 4	11.2%	34.7%	28.5%	11.2%	11.4%	2.5%	0.5%		
**Ethnicity (US)**	White	Black	Asian	Latino	Native	MENA	Mix	Other	Refused
Study 1	70.1%	12.7%	5.5%	3.0%	1.0%	0.2%	6.2%	0.0%	0.7%
Study 2	82.5%	7.5%	1.9%	3.3%	0.6%	0.6%	3.6%	0.0%	0.0%
Study 4	70.1%	6.8%	8.9%	5.9%	0.7%	0.0%	7.1%	0.2%	0.2%
**Ethnicity (Dutch)**	Both parents native-born	One parent foreign-born	Both parents foreign-born		Refused				
Study 3	87.8%	7.2%	4.1%		0.9%				

*Note*. Cloud refers to Cloud Research (formerly called TurkPrime), a crowd funding platform that draws participants from Amazon’s Mechanical Turk (MTurk). Participants from previous studies were excluded from later studies. Exclusions were on the basis of failure of the attention check, with participants in the acceptance or tolerance conditions who indicated that they rarely or never received the ball during the game being excluded, and those in the rejection condition who indicated receiving the ball equally or frequently being excluded. Exclusions were approximately equal in all of the studies, with the exception being Study 3. In Study 3, 22 of the exclusions were from the acceptance condition, 18 from the tolerance condition, and 12 from the rejection condition. Ethnicity was measured differently in the Netherlands (Study 3), where it was assessed by whether parents were born in the Netherlands or abroad. ‘Native’ refers to Native American and Native Hawaiian or Pacific Islander. ‘MENA’ stands for “Middle-East and North Africa”.

## Study 1

### Method

#### Participants

We pre-registered our study design, research questions, and measures at the Open Science Framework (https://osf.io/34dve/?view_only=465c2c25a54848f7a233f5d1a48bae46). However, this was filed before we made last-minute changes to our measures (specifically adding additional items to the well-being measure and including the future behaviour measure). For our first study, we recruited 413 participants using the Cloud Research (formerly TurkPrime) platform for Amazon’s Mechanical Turk (MTurk). Of this total, 11 were excluded from analyses for incorrectly indicating how much they had been included or excluded from the cyberball game, with these exclusions being approximately evenly distributed across the three conditions. Participants were majority white (70.1%), and were mostly between the ages of 26–45 (65.7%). See Table 1 for detailed sample information for all four studies. In the absence of good priors of expected effect size, we conducted the different studies aiming for at least 120 participants per condition. Using the parameters of our smallest sample of 360 participants, along with α = .05 and power of .80, we achieved sufficient sensitivity to consistently find at least small to medium effects, η_p_^2^ = .026.

#### Materials and procedure

*Manipulation*. The goal of designing a novel methodology was to create an environment where we could realistically manipulate and test the effects of being tolerated versus being accepted and rejected. As acceptance, rejection, and tolerance reflect three different combinations of attitude and behavior (see Table 2), the experiment was designed to create those experiences while maintaining a believable cover story for participants to generate interpretable data. The randomized experimental design thus had two primary goals: (a) that participants experience being a minority group member, and (b) that participants believe that their virtual workgroup teammates appreciate and include them (acceptance condition), disapprove of them but nevertheless include them (tolerance condition), or object to and exclude them (rejection condition). To accomplish these goals, participants first completed a set of eight items purportedly designed to test their personality style as a people- vs. task-oriented person, after which they all received bogus feedback that they were people-oriented. Next, participants were shown a set of three comics depicting a people-oriented character either accepting, tolerating, or rejecting a task-oriented character and asked which of the three best matched their preferred approach. Then they were introduced to their three alleged workgroup teammates who were all task-oriented, leaving the participant in a minority position, and who all had supposedly indicated that they accepted (appreciate and include), tolerated (disapprove of but include), or rejected (object to and exclude) people-oriented teammates, such as the participant, for the task ahead. Finally, participants engaged in a cyberball game, based on the classic ostracism manipulation [9, 20], where they were asked to imagine being together in real life with their teammates while passing a ball between them. Both tolerance and acceptance involve behavioral inclusion and therefore in the tolerance and acceptance conditions, the participant was behaviorally fully included in the game. In the rejection condition, the participant was fully excluded after a first round of ball throwing. Thus, we created an online setting to mirror a real-world (virtual) team situation where people might feel and experience acceptance, tolerance, or rejection by having interactions with majority outgroup participants who either accepted their diversity (being people-oriented among task-oriented people), tolerated their diversity, or rejected it (see S1 File for full measures for all studies).

**Table 2:** Manipulating the experience of being accepted, tolerated, and rejected using a novel ostracism paradigm through attitudinal and behavioral components.

	Attitude to Other	Behavior toward Other
Acceptance	Positive and Inclusive	Full Inclusion
Tolerance	Disapproving but Begrudgingly Inclusive	Full Inclusion
Rejection	Negative and Exclusive	Full Exclusion

*Note*. The ‘attitude to other’ was communicated through the text of the comic that each of the participant’s teammates identified with, and the ‘behavior toward other’ was communicated through inclusion or exclusion in the cyberball game.

*Attention check*. To ensure that participants were paying attention during the experiment, at the end of all five studies, we asked participants if they remembered how frequently they were passed the ball in the cyberball game, and excluded participants (2.7% in Study 1) who identified their inclusion level incorrectly (see Table 1 for more details).

*Manipulation check*. At the end of the study participants were asked to indicate whether they felt fully accepted, completely rejected, or tolerated during the study to measure the success of the manipulations. All participants indicated their levels of acceptance, tolerance, and rejection on three separate variables.

*Outcome variables*. We focused on three primary outcomes which matched onto the personal, interpersonal, and raising voice implications of being tolerated, as proposed theoretically [6]: Participants’ personal feelings of well-being during the experimental game, participants’ interpersonal future expectations of how they will be treated and appreciated by their team, and their willingness to complain or take action against their (mis)treatment. Unless otherwise indicated, all items were on 7-point scales.

*Well-being*. We measured personal well-being and identity-need fulfillment drawing on items from previous ostracism [22] and tolerance [7] research. We adapted items that measured feelings of belongingness, sense of control, self-esteem, and meaningfulness in an 11-item measure (see S1 File for complete measures), which formed a single reliable scale (*M* = 3.44; *SD* = 1.84; α = .96). Note that while the measure consisted of both negatively- and positively valanced items, factors scores have been calculated such that higher scores indicate better well-being and lower scores worse well-being.

*Future expectations*. To assess the consequences of tolerance on interpersonal perceptions of being valued or appreciated in future encounters with their teammates, participants were presented with a hypothetical situation in which they would offer a person-oriented solution to a problem the team faces. Four items were presented, asking how open-minded their teammates would be to their suggestion, and how much their teammates would value, incorporate, and solicit their suggestion, which were combined into a single scale (*M* = 4.26; *SD*s = 1.68; α = .96).

*Raising voice*. Raising one’s voice can vary from faint grumbling about one’s discontent to overt complaints and demanding change [14, 15]. In the present context of a behavioral game, we chose to use a subtle measure of minority voice to test the hypothesis that participants in the tolerance condition would be relatively (compared to rejection) unwilling to challenge their devaluation, based on previous criticisms that tolerance may make people less willing to criticize the dominant (working) culture toward a more inclusive and accepting one [17]. In Study 1, we accessed willingness to raise voice by including an open-ended item where participants were asked, “We understand that you were randomly assigned to a team and did not get to choose your fellow team members. Please let us know here what that experience was like for you or if you had any problems with any of your group members as we work to improve this research.” Responses were blind-coded as either 0 or 1, with 0 indicating no complaint and 1 indicating a complaint (*M* = 0.25; *SD* = 0.43).

### Additional variables

#### Emotions

Immediately following the cyberball game, we asked participants to indicate their agreement with emotional statements about how they felt during the game, with three items indicating positive emotions (Motivated, Inspired, Happy; *M* = 3.86; *SD* = 1.94; α = .91) and three items indicating negative emotions (Upset, Nervous, Sad; *M* = 2.10; *SD* = 1.48; α = .83).

#### Personal teamwork beliefs

We then measured participants’ views of themselves as part of a work team with three items (e.g., I believe I can succeed in an online teamwork environment; *M* = 5.51; *SD* = 1.36; α = .89).

#### Trust game

To maintain the cover story, we also developed three items that would appear to be a teamwork-related interactive exercise between the participants and their supposed teammates. We adapted prisoner’s dilemma situations for a work environment to develop items where the ‘best’ outcome would occur when, without the opportunity for consultation, the participant and their supposed teammate would trust each other to make a choice in the interest of both. While these items were not intended for analysis, but were instead created to sustain the cover story, we nonetheless analysed them by combining the three items to form a 4-point (0–3) scale, with 1 point assigned for each of three decisions where a participant gave a trusting response (*M* = 2.08; *SD* = 0.89).

*Analyses*. All analyses were conducted using SPSS and the datasets can be accessed along with study materials on the Open Science Framework (https://osf.io/ad9ec/?view_only=4c1fdf1b8bfa4c639432399f8e5d8698). While descriptive statistics are reported using means derived from averaging items together to form a scale, inferential statistics have been conducted using factor scores where applicable.

### Results

#### Manipulation check

First, we tested whether participants experienced the study differently based on their condition. We measured participants’ feelings of being accepted, tolerated, and rejected using three items. We found significant condition differences for the rejection, *F*(2, 398) = 353.69, *p* < .001, η_p_^2^ = .640, tolerance, *F*(2, 398) = 72.99, *p* < .001, η_p_^2^ = .268, and acceptance, *F*(2, 398) = 317.96, *p* < .001, η_p_^2^ = .615, items, with all Tukey-corrected comparisons indicating significant differences (*p* < .05) between conditions.

As Table 3 shows, for the feeling of acceptance, we found that people in the acceptance condition felt most accepted, followed by those in the tolerance condition, and finally those in the rejection condition. For the feeling of rejection, we found that people in the rejection condition felt it most, followed by those in the tolerance condition, and finally those in the acceptance condition. The results for the tolerance item were a little unexpected, as we found that people in the acceptance condition felt most tolerated, followed by those in the tolerance condition, and finally those in the rejection condition. As we explain further below, we believe this to be due to confusion people may have about the meaning of the term tolerance when asked about it directly. Indeed, this was also the reason for an experimental paradigm which avoided the problem of individual understandings of what tolerance is.

**Table 3:** Results of the manipulation check items for Study 1.

	Experimental conditions		
	Rejection	Tolerance	Acceptance
Feeling Rejected	6.11 (1.65)_a_	2.33 (1.65)_b_	1.54 (1.15)_c_
Feeling Tolerated	1.70 (1.20)_a_	3.12 (2.00)_b_	4.35 (2.09)_c_
Feeling Accepted	1.70 (1.31)_a_	4.98 (1.51)_b_	5.75 (1.36)_c_

*Note*. Different subscripts indicate significant mean differences (*p* < .05) across the rows (calculated with Tukey post-hoc correction).

### Primary outcomes

#### Well-being

Personal well-being was strongly affected by whether participants experienced acceptance, tolerance, or rejection, *F*(2, 398) = 301.50, *p* < .001, η_p_^2^ = .602. As expected (H1), that well-being was lowest among those who were rejected, followed by those who were tolerated, and with the most positive well-being among those who were accepted (the latter two also being significantly different from each other; see Table 4).

**Table 4:** Key outcome variable contrasts for Study 1.

	Experimental conditions				
	Rejection	Cohen’s *d*	Tolerance	Cohen’s *d*	Acceptance
Well-being	-1.09 (.703)_a_	2.14	.378 (.639)_b_	-.53	.694 (.547)_c_
Future expectations	-.548 (1.05)_a_	.58	.019 (.887)_b_	-.61	.518 (.751)_c_
Raising Voice	.647 (.480)_a_	-1.40	.091 (.289)_b_	.30	.022 (.147)_b_

*Note*. Analyses on well-being and future expectations were performed on factor scores, while collective action was a dichotomous coding. Different subscripts signify significantly different mean scores across the rows (at *p* < .05; calculated with Tukey post-hoc correction). Cohen’s *d* columns indicate the effect size between the two conditions (rejection and tolerance, tolerance and acceptance) calculated from the non-standardized means.

#### Future expectations

Participants showed a similar pattern of effects in their expectations of how they would be treated in interpersonal future interactions with their teammates, and how much their perspectives and ideas would be valued and sought out, *F*(2, 398) = 47.04, *p* < .001, η_p_^2^ = .191. As expected (H2), those in the rejection condition had the lowest expectations of valued interactions and those in the acceptance condition showed the highest, with those who were tolerated falling in between the two (all conditions significantly different from each other; see Table 4).

#### Raising voice

*T*he coded variable measuring whether or not participants complained about their devaluation when offered the chance, also revealed significant effects, *F*(2, 398) = 140.94, *p* < .001, η_p_^2^ = .415, which supported the argument that tolerance could have depoliticizing effects (H3). While we wouldn’t, of course, expect those who were tolerated to feel as strongly about the need to take action as those who were rejected, we expected that they would feel sufficiently strongly to take some action to improve a situation in which one is disapproved of which has negative personal and interpersonal consequences, compared to being fully accepted. However, those who were tolerated were as unlikely to complain as those who were fully accepted. This suggests a depoliticizing effect in which being tolerated does not increase the willingness to complain despite the lower wellbeing and negative future expectations.

#### Additional variables

For both positive, *F*(2, 398) = 162.27, *p* < .001, η_p_^2^ = .450, and negative, *F*(2, 398) = 40.65, *p* < .001, η_p_^2^ = .170, emotions following the game, results indicated main effects of the cyberball portion of the experiment alone (where those in the rejection condition were excluded while those in both tolerance and acceptance were fully included), such that the acceptance (*M*_*PositiveEmotions*_ = 0.51; *SD* = 0.75; *M*_*NegativeEmotions*_ = -0.38; *SD* = 0.57) and tolerance (*M*_*PositiveEmotions*_ = 0.43; *SD* = 0.81; *M*_*NegativeEmotions*_ = -0.19; *SD* = 0.85) conditions differed from rejection (*M*_*PositiveEmotions*_ = -0.95; *SD* = 0.67; *M*_*NegativeEmotions*_ = 0.58; *SD* = 1.21), which may have been due to the wording of the items that specifically asked about participants’ feelings during the ball-passing game, where only those in the rejection condition had a different experience than those in both acceptance and tolerance conditions.

A similar pattern emerged for the items on personal teamwork beliefs, *F*(2, 398) = 19.39, *p* < .001, η_p_^2^ = .089, with the acceptance (*M* = 0.30; *SD* = 0.82) and tolerance (*M* = 0.09; *SD* = 0.84) conditions differing from rejection (*M* = -0.41; *SD* = 1.17).

Finally, the exploratory analysis of the prisoner’s dilemma-style trust items revealed a weak effect, *F*(2, 398) = 7.63, *p* = .001, η_p_^2^ = .037, with acceptance (*M* = 2.29; *SD* = 0.86) differing from rejection (*M* = 1.87; *SD* = 0.92), but neither differing from tolerance (*M* = 2.08; *SD* = 0.83) which fell in between.

### Discussion

The results from Study 1 offer a promising direction to experimentally examine the belief of being tolerated. Across three key variables measuring the personal (well-being), interpersonal (future expectations of treatment), and voice consequences of tolerance, the predicted effects emerged: while the begrudging inclusion of tolerance is much better than being rejected, it nonetheless does not provide all of the benefits of being fully accepted. Specifically, we showed with both well-being (H1) and future expectations (H2) that being tolerated (compared to acceptance) can degrade participants’ well-being and self-perceptions as well as make them feel that they are unlikely to be fully welcomed in future encounters. This effect is noteworthy because it surpasses the established and strong behavioral ostracism effect of cyberball: Those who experienced tolerance were as fully included in the actual cyberball game as those in the acceptance condition and it was the simple awareness that their teammates were begrudgingly including them that made a difference. Furthermore, we also found evidence for the depoliticization hypothesis (H3) despite the measurable negative consequences of being tolerated: tolerated minorities can be disinclined from expressing their displeasure about the devaluation, even as they feel worse and less welcome than when being fully accepted.

However, Study 1 had some possible shortcomings and should be extended for reasons of conceptual replication [23]. First, while the results in terms of outcome variables supported most of our predictions, the manipulation check item asking the extent to which participants felt tolerated, was interpreted in such a way that those in the acceptance condition felt more tolerated than those in the tolerance condition. As we noted, this could be due to people having difficulties in identifying an experience as being tolerated [see 8, 9], which, in itself was one of the reasons for developing a tolerance manipulation to begin with. However, this suggested to us that in order to have a manipulation check measure, the wording of the item need to be improved.

Second, while the well-being measure was adapted from previous cyberball research, the selection of items did not fully capture the range of well-being components. Therefore, we adapted a number of the items in Study 2 to improve the measure, while still aiming to capture a personal measure of well-being.

Third, while open-ended responses of raising voice offered more participant-driven indications of willingness to complain, leaving the measurement up to dichotomous coding left room for error. Therefore, the raising voice measure was adapted into a closed-response measure in Study 2.

Fourth, the results of Study 1 indicated that the placement and wording of the emotion items meant that they functionally served as a manipulation check on the cyberball aspect of the experiment. Nonetheless, we decided that having an emotional response about the ball-passing activity alone was useful as both a check on the measure and to sustain the cover story, so the items were retained and expanded to eight items for Study 2. We maintained the trust items for the same reason.

Fifth, given the limited findings for the generally-worded measure of personal teamwork beliefs, we opted to remove that measure for Study 2 and replaced it with a more specific measure of expected future withdrawal in interpersonal situations.

## Study 2

### Method

#### Participants

For Study 2, we recruited 364 participants from Cloud Research and MTurk, with four exclusions leaving 360 for analysis (see Table 1 for demographics).

#### Materials and procedure

The materials for Study 2 were mostly identical to those of Study 1, with no changes made to the experimental materials themselves or the attention check item. The wording of the manipulation check was slightly adapted from Study 1 in an attempt to clarify what we meant by tolerance by emphasizing the negativity element, that tolerance is when someone disapproves of you but nonetheless does not exclude you. For well-being, the items were slightly changed to better reflect a theoretical range of well-being components to form a 12-item scale (*M* = 3.72; *SD* = 1.68; α = .95; see S1 File). The measure for future expectations was the same as in Study 1 (*M* = 3.87; *SD* = 1.57; α = .95), and an additional 4-item scale of social withdrawal from the team was added asking about how participants thought they would respond in future interactions (*M* = 3.14; *SD* = 1.20; α = .71). The raising voice measure was adapted from an open-ended item into a two-item closed-ended set with the first asking if participants experienced any uncooperativeness or problems and if they would recommend that any of their teammates be excluded from future studies (*M* = 1.93; *SD* = 1.10; *r* = .62). Finally, the trust items were measured and scored the same way as in Study 1 on a 4-point scale (*M* = 2.01; *SD* = 0.82).

### Results

#### Manipulation check

The results of manipulation check were again mostly consistent with our expectations. Those in the acceptance condition reported feeling more accepted (*M* = 5.38; *SD* = 1.35) than those in the tolerance condition (*M* = 4.32; *SD* = 1.77), who in turn felt more accepted than those in the rejection condition (*M* = 1.84; *SD* = 1.41), *F*(2, 357) = 170.12, *p* < .001, η_p_^2^ = .488. Further, those in the rejection condition felt more rejected (*M* = 5.96; *SD* = 1.53) than those in the tolerance condition (*M* = 2.16; *SD* = 1.59), and those in the acceptance condition (*M* = 1.62; *SD* = 1.03), *F*(2, 357) = 341.02, *p* < .001, η_p_^2^ = .656. However, the more negative wording of the tolerance item seems not to have worked as intended, with those in the acceptance condition again reporting the most tolerance (*M* = 5.64; *SD* = 1.57), followed by the tolerance condition (*M* = 2.95; *SD* = 1.76), and in turn followed by the rejection condition (*M* = 1.87; *SD* = 1.17), *F*(2, 357) = 196.63, *p* < .001, η_p_^2^ = .524.

#### Primary outcomes

Results for the primary personal (well-being), interpersonal (future expectations and future withdrawal), and voice (raising voice) outcomes were all consistent with Study 1 and in support of H1, H2, and H3. Well-being was again lowest in the rejection condition, followed by the tolerance condition, with the highest well-being reported in the acceptance condition, *F*(2, 357) = 237.93, *p* < .001, η_p_^2^ = .571 (all conditions were significantly different from each other, see Table 5). Similarly, future expectations of teammate treatment were most positive in the acceptance condition, followed by the tolerance condition, and then in the rejection condition, *F*(2, 357) = 84.93, *p* < .001, η_p_^2^ = .322 (all conditions were significantly different from each other).

**Table 5:** Key outcome variable contrasts for Study 2.

	Experimental conditions				
	Rejection	Cohen’s *d*	Tolerance	Cohen’s *d*	Acceptance
Well-being	-1.04 (.678)_a_	1.90	.321 (.741)_b_	-.63	.721 (.534)_c_
Future expectations	-.621 (.964)_a_	.55	-.130 (.825)_b_	-1.18	.750 (.659)_c_
Future withdrawal	.354 (1.19)_a_	.37	-.010 (.872)_b_	-.40	-.344 (.767)_c_
Raising voice	2.89 (1.01)_a_	-1.44	1.54 (.853)_b_	.24	1.35 (.672)_b_

*Note*. Analyses on well-being, future expectations, and future withdrawal were performed on factor scores. Different subscripts signify significantly different mean scores across the rows (at *p* < .05; calculated with Tukey post-hoc correction). Cohen’s *d* columns indicate the effect size between the two conditions (rejection and tolerance, tolerance and acceptance) calculated from the non-standardized means.

Additionally, those in the tolerance condition, despite reporting negative effects of being tolerated compared to being accepted, were again not more likely to raise their voice to protest against their begrudging treatment compared to those who were fully accepted, *F*(2, 357) = 114.43, *p* < .001, η_p_^2^ = .391. This again suggests that being tolerated, despite its negative consequences, seems to be dampening action for improvement, consistent with the depoliticization critique of tolerance in diverse settings [6, 17]. The new interpersonal variable measuring how participants thought they would behave in a future encounter with their team was also statistically significant, *F*(2, 357) = 15.88, *p* < .001, η_p_^2^ = .082. The more interactive behavior was expected by those in the acceptance condition, followed by those in the tolerance condition, and finally followed by those in the rejection condition (all conditions significantly different from each other; Table 5).

#### Other variables

The emotion measures in Study 2 did not load into separate positive and negative scales, but rather all of the emotions, aside from dependence, loaded onto one positive emotion scale, on which we found, again, the exclusion effect on positive emotions, *F*(2, 357) = 251.736, *p* < .001, η_p_^2^ = .585, such that positive emotions did not differ between the tolerance (*M* = .467, *SD* = .607) and acceptance (*M* = .611, *SD* = .571) conditions, but both differed from the rejection (*M* = -1.08, *SD* = .751) condition. The single-item dependence emotion showed no effects, *F*(2, 357) = 1.634, *p* = .197, η_p_^2^ = .009, such that dependence did not differ between any of the conditions: tolerance (*M* = .140, *SD* = .091), acceptance (*M* = -.048, *SD* = .091) and rejection (*M* = -.073, *SD* = .091).

The prisoner’s dilemma-style trust game, constructed primarily to sustain the experimental cover story, showed no significant effects, *F*(2, 357) = .775, *p* = .462, η_p_^2^ = .004, such that trust did not differ between the tolerance (*M* = 2.03, *SD* = .788), acceptance (*M* = 2.06, *SD* = .792), and rejection (*M* = 1.93, *SD* = .886) conditions.

### Discussion

The results of Study 2 provided additional support for the three predictions and the main conclusions we arrived at with Study 1. First, the experimental methodology offers a consistent and believable way to give participants the experience of being tolerated (compared to being accepted or rejected) in an online working context. Second, compared to being fully accepted, the tolerance experimental experience of being begrudgingly included leads to decreased well-being (H1), more negative perceptions of future treatment and higher expectations of withdrawal (H2); however, tolerance is still a much less negative experience than being rejected. Third, we again found evidence for depoliticization, with those who were begrudgingly included being no more willing than those who were fully accepted to protest their treatment (H3).

Study 2 showed that the wording of the tolerance manipulation check item did not overcome what we believed to be confusion when it comes to direct measures of the concept. Tolerance is sometimes conceptualized as meaning open-minded and fully accepting [6], so participants may have trouble understanding what the item is referring to especially when the item has no built-in comparative anchors. Therefore, for Study 3 we opted for an item that placed the experience of tolerance in the middle of a continuum from rejection to acceptance experiences as a manipulation check.

Studies 1 and 2 also had two other limitations we aimed to address with Study 3. First, both studies were conducted with convenient online samples which can raise questions about the reliability and validity of the findings [24, but see 25]. Second, both studies used American participants and it would increase confidence in the strength of the phenomenon if these findings replicate to another country and language. Therefore, in Study 3 we adapted the experiment for application to a national sample in the Netherlands.

## Study 3

### Method

#### Participants

Using Dutch survey company PanelInzicht, we recruited 610 participants from across the Netherlands to participate in a Dutch language translation of the study, with 52 participants excluded for failing to correctly identify how often they received the ball in the cyberball portion of the study (the same exclusion criteria used in Studies 1 and 2; see Table 1).

#### Materials and procedure

The materials for Study 3 were nearly identical to those of Study 2, with the only change being to the manipulation check which was adapted into two items asking how participants felt during the team exercises using 11-point scales from fully rejected (1) to tolerated (6) to fully accepted (11).

Personal well-being (*M* = 3.47; *SD* = 1.26; α = .92), future expectations (*M* = 4.26; *SD* = 1.26; α = .91), participants’ future withdrawal (*M* = 3.15; *SD* = 1.15; α = .74), and willingness to raise voice (*M* = 2.08; *SD* = 1.00; *r* = .60), were all identical to Study 2.

The items retained to sustain the cover story which measured negative and positive emotions during the ball-passing portion and the prisoners’ dilemma trust game were also included and findings are presented below.

### Results

As Table 6 shows, results of the national Dutch sample closely mirrored those of the American sample, although effects were almost always smaller than in the American convenience samples.

**Table 6:** Key outcome variable contrasts for Study 3.

	Experimental conditions				
	Rejection	Cohen’s *d*	Tolerance	Cohen’s *d*	Acceptance
Well-being	-.576 (1.01)_a_	.81	.177 (.878)_b_	-.30	.426 (.785)_c_
Future expectations	-.286 (1.07)_a_	.32	.035 (.974)_b_	-.34	.300 (.798)_c_
Future withdrawal	.036 (1.06)_a_	.04	.072 (1.02)_a_	-.23	-.103 (.943)_a_
Raising voice	2.72 (.931)_a_	-.90	1.90 (.894)_b_	.42	1.55 (.753)_c_

*Note*. Analyses on well-being, future expectations, and future withdrawal were performed on factor scores. Different subscripts signify significantly different mean scores across the rows (at *p* < .05; calculated with Tukey post-hoc correction). Cohen’s *d* columns indicate the effect size between the two conditions (rejection and tolerance, tolerance and acceptance) calculated from the non-standardized means.

#### Manipulation check

The new manipulation check items performed as we intended, as all three conditions were distinct from each other, *F*(2, 555) = 94.90, *p* < .001, η_p_^2^ = .255. Specifically, those in the acceptance condition indicated relatively higher levels of acceptance (*M* = 8.29, *SD* = 1.63) than those in the rejection condition (*M* = 5.39, *SD* = 2.57), and those in the tolerance condition (*M* = 7.58, *SD* = 2.07), while those in the rejection condition indicated less perceived acceptance (and more perceived rejection) than those in the tolerance condition.

#### Primary outcomes

Once again, well-being was lowest in the rejection condition, followed by the tolerance condition, and the highest in the acceptance condition, *F*(2, 555) = 63.94, *p* < .001, η_p_^2^ = .187, with all conditions significantly different from each other (H1).

Similarly, the same patterns as in previous studies emerged for future expectations of the teammates’ treatment of the participants, *F*(2, 555) = 17.48, *p* < .001, η_p_^2^ = .059. Unlike Study 2, however, the new measure of future withdrawal did not differ across condition, *F*(2, 555) = 1.51, *p* = .223, η_p_^2^ = .005.

For raising voice, in Study 3 we found that being tolerated did result in a greater willingness to complain compared to those in the acceptance condition, contrary to the depoliticization findings of the other studies, with rejection once again showing the greatest willingness to complain, *F*(2, 555) = 90.38, *p* < .001, η_p_^2^ = .246 (see Table 6).

#### Other variables

Emotions after the cyberball game again showed the cyberball exclusionary effect, *F*(2, 555) = 26.981, *p* < .001, η_p_^2^ = .089, such that negative emotions did not differ between the tolerance (*M* = -.137, *SD* = .811) and acceptance (*M* = -.286, *SD* = .900) conditions, but both differed from the rejection (*M* = .390, *SD* = 1.08) condition. The same effect appeared for positive emotions, *F*(2, 555) = 137.648, *p* < .001, η_p_^2^ = .332, such that positive emotions did not differ between the tolerance (*M* = .432, *SD* = .749) and acceptance (*M* = .390, *SD* = .805) conditions, but both differed from the rejection (*M* = -.786, *SD* = .883) condition.

Once again, no effect emerged for the Trust filler game, *F*(2, 555) = 1.978, *p* = .139, ηp^2^ = .007, such that trust did not differ between the tolerance (*M* = 1.76, *SD* = .808), acceptance (*M* = 1.80, *SD* = .748), and rejection (*M* = 1.65, *SD* = .797) conditions.

### Discussion

Study 3 conducted among a national sample in the Netherlands broadly confirmed the findings that we found among convenient U.S. samples and our predictions. This further enhances our confidence in the generalizability of the proposed theoretical expectations about the impact of being tolerated on well-being and perceptions of future interactions. However, there were some differences worth noting. First, the depoliticization effect differed somewhat from the previous two studies with those who were tolerated indicating a greater willingness to complain than those who were accepted. Second, the new variable measuring future behavioral expectations did not replicate in the Dutch sample. Further, we should note that effects overall were weaker in the Dutch sample than in the American samples, which may be due to unattended selective attrition in online convenience samples such as MTurk [24]. There might also be relevant cultural differences between the United States and the Netherlands, such as the Netherlands being a more egalitarian country and the relatively higher level of support in the US for individualistic values [26, 27]. Nonetheless, with this study we were again able to provide additional evidence for the novel methodology to experimentally manipulate feelings of being begrudgingly included, as well as evidence about the unintended negative effects of this approach to managing diversity.

## Study 4

In a fourth study, we aimed to dig deeper into the question of depoliticization. While Studies 1 and 2 showed evidence of depoliticization, we did not find the same effect in Study 3. Therefore, in Study 4, we decided to test the depoliticization hypothesis (H3) using a more robust behavioral index of raising one’s voice by using a (bogus) public review website where participants could rate and report their experiences in the online team study and whether they recommend the exercise to other people. By having participants respond external to the study and in the supposed view of the online public, we aimed to better capture the willingness to raise one’s voice and take action. Thus, this measure of depoliticization was intended to more closely approximate real-world behavior.

### Method

#### Participants

We recruited 450 participants from MTurk for this study, of which 12 were excluded for failing the attention check, leaving 438 participants for analysis (see Table 1 for demographics; also see Open Science Framework for pre-registration of the data collection method for the novel ‘public’ measure of depoliticization: https://osf.io/xbndk/?view_only=9cc46e83abff47748ddb937c4a12473f).

#### Materials and procedure

The materials for Study 4 were nearly identical to those of Study 3, with an additional measure of raising voice which we discuss in more detail below. Well-being (*M* = 3.63; *SD* = 1.69; α = .95), future expectations of teammate treatment (*M* = 3.95; *SD* = 1.63; α = .95), expectations of personal behavior in the team (*M* = 3.02; *SD* = 1.30; α = .78), and the close-ended items willingness to raise voice (*M* = 1.94; *SD* = 1.10; *r* = .55), were all identical to Study 3, and the cover story items were again retained in the survey design and presented in the analysis.

*Public expression of minority voice*. Study 4 used a bogus review website to investigate the effects of feeling tolerated, compared to being accepted and rejected, on minority voice by examining how these differing experiences impact on one’s willingness to publicly post about one’s experiences. We measured the extent to which participants were willing to provide public feedback ratings and post recommendations for participation in the study. For the sake of realism, we encouraged, but did not require, participants to give public feedback on a separate webpage to which they were redirected at the conclusion of the study.

### Results

#### Manipulation check

Once again the manipulation check confirmed distinct experiences across the three conditions, *F*(2, 435) = 444.47, *p* < .001, η_p_^2^ = .671, with those in the acceptance condition being the closest to the acceptance end of the scale (*M* = 9.26; *SD* = 1.74), those in the rejection condition falling close to the rejection end of the scale (*M* = 2.74; *SD* = 2.35), and those in the tolerance condition significantly different from both acceptance and rejection, though closer to the acceptance side of the scale (*M* = 8.29; *SD* = 1.88).

#### Primary outcomes

The results for personal well-being (H1), *F*(2, 435) = 306.66, *p* < .001, η_p_^2^ = .585, future expectations (H2), *F*(2, 435) = 104.63, *p* < .001, η_p_^2^ = .325, and willingness to raise voice in protest (H3), *F*(2, 435) = 160.11, *p* < .001, η_p_^2^ = .424, were consistent with the findings of previous studies (again finding the depoliticization effect as in Studies 1 and 2). This reaffirms the negative consequences of being tolerated relative to acceptance, and strengthens the case of a depoliticization effect of tolerance. Once again, the added measure of expectations of future withdrawal did not show any difference between the tolerance and acceptance conditions, *F*(2, 435) = 26.33, *p* < .001, ηp^2^ = .108 (see Table 7).

**Table 7:** Key outcome variable contrasts for Study 4.

	Experimental conditions				
	Rejection	Cohen’s *d*	Tolerance	Cohen’s *d*	Acceptance
Well-being	-1.09 (.696)_a_	2.22	.432 (.660)_b_	-.34	.640 (.584)_c_
Future expectations	-.718 (.915)_a_	.81	-.003 (.846)_b_	-.88	.676 (.700)_c_
Future withdrawal	.454 (1.07)_a_	.60	-.147 (.949)_b_	-.20	-.320 (.823)_b_
Raising voice	2.98 (.938)_a_	-1.85	1.43 (.710)_b_	-.03	1.46 (.844)_b_

*Note*. Analyses on well-being, future expectations, and future withdrawal were performed on factor scores. Different subscripts signify significantly different mean scores across the rows (at *p* < .05; calculated with Tukey post-hoc correction). Cohen’s *d* columns indicate the effect size between the two conditions (rejection and tolerance, tolerance and acceptance) calculated from the non-standardized means.

*Public expression of minority voice*. To delve more deeply into the potential depoliticizing effects of tolerance, we looked at the data for the public-facing measure of taking action through raising voice to complain about the experience. As we had decided to make this optional to sustain the cover story and to give participants a feeling of distance from the study they chose to participate in, we first looked at participation rate. Of 438 total participants who had the option of posting publicly on the website we created for the study, 318 (72.6%) left at least partial feedback on the bogus website, with similar response rates in the rejection (104; 73.76%), tolerance (104; 71.23%), and acceptance (110; 72.85%) conditions.

Participants were asked to post on the website whether they recommend the study or not using a thumbs-up or thumbs-down option, and we found evidence for depoliticization on this measure, as people who were tolerated (95 recommendations in favor, 9 against; *M* = .914, *SD* = .283) were not more willing to vote the study down than those who were fully accepted and had no reason to vote the study down (102 recommendations in favor, 8 against; *M* = .927, *SD* = .261). Unsurprisingly, those who were rejected were the most willing to vote the study down (77 recommendations in favor, 27 against; *M* = .740, *SD* = .441), *F*(2, 315) = 10.09, *p* < .001, η_p_^2^ = .060 (with those in the tolerance condition showing a medium difference from those who were rejected, Cohen’s *d* = .47, and a small difference from those who were accepted, Cohen’s *d* = .05).

The same pattern of depoliticization emerged when we analyzed the factor score of three attitude questions on the ‘public’ ratings webpage, *F*(2, 279) = 46.65, p < .001, η_p_^2^ = .251. People who were tolerated (*M* = .296, *SD* = .769) were equally positive as those who were accepted (*M* = .391, *SD* = .723), and both gave more positive ratings than people who were rejected (*M* = -.714, *SD* = 1.07), with small differences between the tolerance and acceptance conditions, Cohen’s *d* = .13, and large differences between tolerance and rejection, Cohen’s *d* = 1.08. Thus, even using the more robust and public-facing measure of raising one’s voice, we again find evidence for the depoliticizing effect of tolerance: the negative personal and interpersonal consequences of being begrudgingly included does not generate action to improve how the tolerated are treated.

#### Other variables

As in Study 2, the emotion measures again comprised a single scale of all items aside from dependence, *F*(2, 435) = 422.134, *p* < .001, η_p_^2^ = .660, with positive emotions not differing between the tolerance (*M* = .507, *SD* = .555) and acceptance conditions (*M* = .608, *SD* = .507), but both differing from experiencing rejection (*M* = -1.18, *SD* = .683). No effects emerged for the single-item dependence emotions, *F*(2, 435) = 1.026, *p* = .359, η_p_^2^ = .005.

The prisoner’s dilemma trust filler game showed that people were more trusting in the tolerance (*M* = 2.15, *SD* = .782) and acceptance (*M* = 2.17, *SD* = .820) conditions, compared to rejection (*M* = 1.87, *SD* = .904), *F*(2, 435) = 5.885, *p* = .003, η_p_^2^ = .026.

### Discussion

Study 4 replicated the personal and interpersonal effects we had found in the previous three studies and across two different national contexts, with being tolerated having more negative effects than being fully accepted, but still being far superior to being rejected. Importantly, the standard measure of raising voice to create change, again showed the depoliticization effect, and we found further support for the depoliticization hypothesis using the robust ‘public website’ measure as well.

## Mini meta-analysis

**Fig 1:**
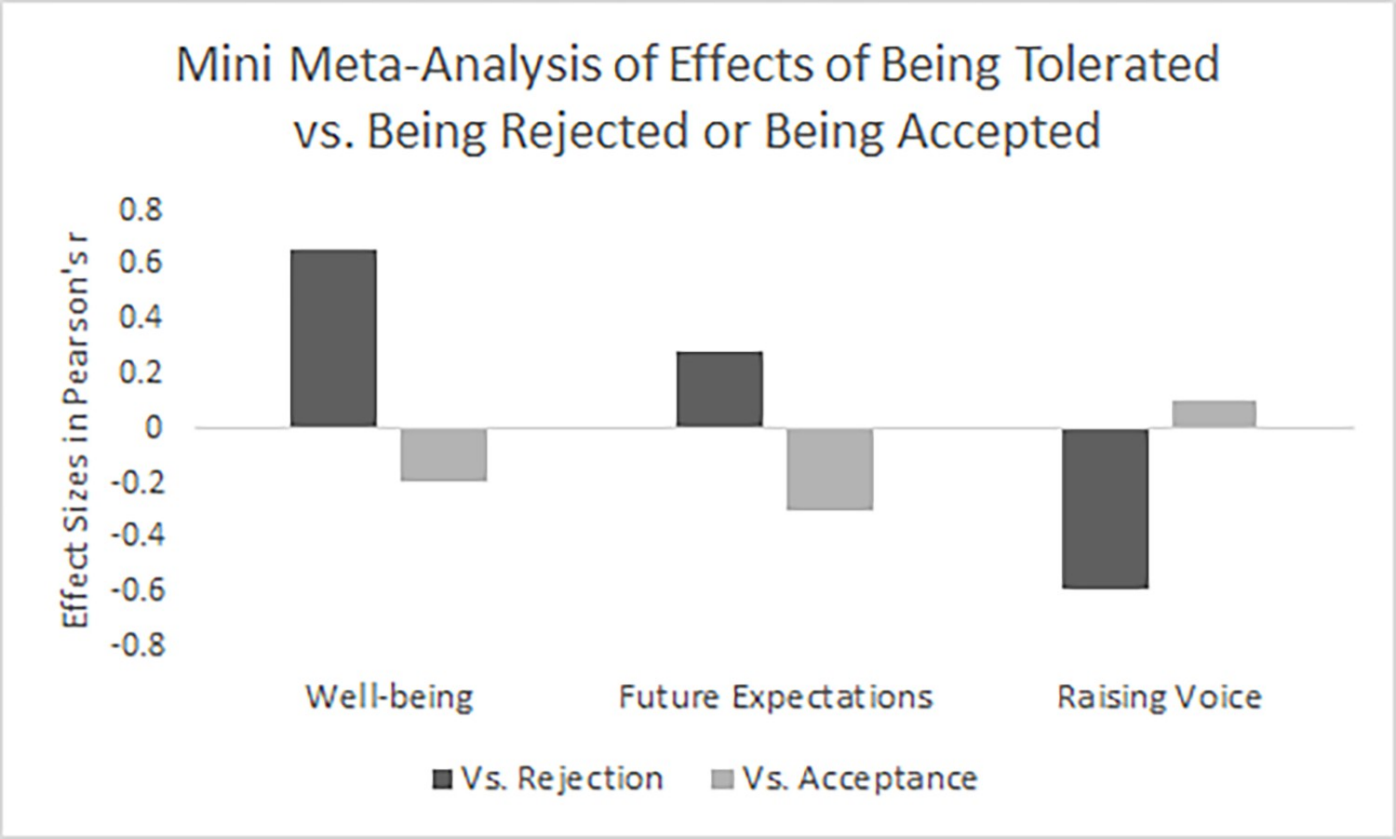
The weighted mean effect sizes of the effect of being tolerated compared to being rejected (dark grey bars) and being accepted (light grey bars) on well-being, future expectations, and raising voice.

To have a better overview of the evidence for the consequences of being tolerated on the expected personal, interpersonal, and voice outcomes across the four studies, we conducted a mini meta-analysis of the results using weighted mean effect sizes [28]. Looking first at whether tolerance led to better outcomes than discrimination, we found that across all of the studies, tolerated people strongly displaying better wellbeing than rejected people (*r* = .66), and reported moderately higher expectations of being treated well by their teammates compared to rejected people (*r* = .28). Unsurprisingly, tolerated people also showed comparatively strong unwillingness to complain about their mistreatment and raise their voice compared to rejected people (*r* = -.59). However, across all four studies, the evidence also shows that tolerance is inferior to being fully accepted, as those who were tolerated displayed weak-to-moderate lower wellbeing (*r* = -.20), and had moderately lower expectations of being treated well by their teammates (*r* = -.30). Importantly, collapsing across all four studies, evidence of depoliticization emerged as an increased willingness to complain and raise voice. However, that willingness represents an effect size two to three times smaller than the negative effects of tolerance on their well-being and future expectations (*r* = .10). Thus, across the four studies, the weight of the evidence indicates that tolerance is better than rejection, but worse than acceptance, with an additional depoliticizing effect (see Fig 1).

## General discussion

Tolerance as forbearance is critically important in many situations because people in diverse organizations and pluralistic societies will inevitably dislike, disagree, or disapprove of some convictions, beliefs and conduct of others [3, 4]. Tolerance is considered a key ingredient in social relations and for diverse settings because people need to endure what they object to in order to let others express their dissenting views, beliefs, and ways of living [2–5]. However, tolerance contains the implication of disapproval and devaluing of the minority beliefs, values, and behaviors. Furthermore, it also has been argued that the experience of being tolerated can have depoliticizing consequences and is inescapably patronizing and therefore an inadequate substitute for the appreciation and full acceptance that minority members seek [6, 17, 18]. Thus, for evaluating the role and impact of forbearance tolerance for diverse settings it is necessary to systematically consider the target’s perspective.

Across four studies in two countries, we experimentally examined the experience of being tolerated on the personal effect of minority well-being, the interpersonal effect of future expectations of treatment by majority group members, and the politicized effect of willingness to raise one’s voice against being tolerated. We expected that being begrudgingly included to be a rather negative experience in comparison to full acceptance, but rather positive compared to being rejected. Focusing on virtual teams which are increasingly common and using a novel methodology based in research on interpersonal and intergroup ostracism [10, 29, 30], allowed us to create a realistic situation and to bypass the problems posed by depending on individual interpretations of the widely used word ‘tolerance’ [3]. As expected, we found that being tolerated indeed had more negative implications than that of being accepted, but being begrudgingly included was also much better for minority group members’ well-being (H1) and expectations of future treatment (H2) than being rejected. Thus, being tolerated had more positive implications than being rejected but it fell short of being fully accepted.

The results for depoliticization indicate that people who were tolerated were no more willing to raise their voice against being begrudgingly included on the basis of their personal style than those who were fully accepted (H3), including in one study where we used a bogus public review page to evaluate participants’ willingness to complain and take action. This is especially noteworthy given the negative effects of being tolerated (compared to acceptance) on well-being and expectations about future treatment.

Our findings demonstrate that the experience of being tolerated is not necessarily a positive one: tolerance contains the message that one’s beliefs and practices are disapproved of and that one is begrudgingly included [6]. We experimentally established these effects in a virtual work team setting following preliminary evidence for the negative implications of being tolerated in survey research among ethnic, sexual and disabled minorities [7–9]. This suggests that being ‘put up with’ is not only challenging for those who may experience this in real life, but also for those experimentally induced to experience it in a virtual team which is a setting that is increasingly an established reality in many companies, organizations and institutions.

Another important implication of the current research is that the differences between being accepted and being tolerated emerged from simple awareness that one’s team members would prefer not to engage with people with their working style, but yet begrudgingly agreed to include them in the team tasks and did so equally in their behavior. While it is unlikely that people might be told this explicitly, members of minority groups and orientations may be primed for the relatively subtle cues of exclusion and othering. This is important because while extensive research demonstrates the psychological effects of behavioral inclusion versus exclusion using the cyberball game, part of our findings emerge from comparing participants who were identically treated during the game but either believed to be begrudgingly included or rather welcomed for their differences.

Furthermore, this finding is also relevant in light of the microaggression literature in which it is proposed that the actual intention or attitude behind the microaggression does not matter for the victim to feel microaggressed [31, 32]. However, the difference in attitude is exactly what matters for the distinction between being tolerated and being accepted. Specific for the experience of being tolerated is that the behavior is inclusive but the attitude is not. Being begrudgingly included, therefore, differs from forms of microaggression and future research might consider to empirically examine these differences more systematically. Further, the experience of being tolerated differs from forms of rejection and discrimination in which victims are behaviorally excluded which has various negative psychological and social implications [11, 12]. Some of the strongest experimental effects that we found were between being rejected and being tolerated which suggests that facing actual negative treatment is much more painful and problematic than knowing or believing that others disapprove of you and endure you while nevertheless treating you fairly.

### Limitations and conclusion

While the current research provides convergent evidence across multiple experiments in two national contexts, it could be argued that being tolerated in all of these situations has negative psychological implications because acceptance of differences tends to be normative within these team settings. The language of diversity and the appreciation of differences have become predominant in Western liberal societies such as the United States and the Netherlands, and in most organizational, institutional and work contexts in these countries [33, 34]. Thus, being begrudgingly included might have negative implications because it is considered rather inappropriate, which is indicated by the use of the term ‘mere tolerance’. Future research could investigate this interpretation, for example, by examining the implications of being tolerated in non-Western, illiberal societies in which the emphasis is much less on individual freedoms and rights, or in an organizational context where appreciation and acceptance of differences is not normative (e.g., an authoritarian organizational culture where hierarchy and inequality are common). In such spaces, we may find more positive responses to tolerance (see [35]). Indeed, historically, persecuted people have shown a distinct preference for tolerance, such as the Jews who fled persecution in Western Europe for the religious tolerance of Poland and the Ottoman Empire. Although they were not fully accepted in the other countries, they sought out tolerance. Furthermore, during the Dutch Revolt in the 16^th^ Century of Dutch protestants against Catholic Spain, some protestant Dutch soldiers wore medallions reading “Better under the Turks than the Pope” indicating a preference for the religious tolerance of the Ottoman Empire over their experience with the Spanish.

Additionally, this interpretation also implies that the impact of being tolerated can be different in contexts or in relation to differences for which forbearance tolerance rather than appreciation is normative. For example, forbearance tolerance rather than appreciation is likely to be normatively expected when it comes to different ideological viewpoints and political opinions. And whereas people may expect to be accepted despite their different working style (person oriented), someone who is less conscientious or who expresses a lack of motivation for the team’s objective might expect tolerance at best. Thus, there might be contexts and differences for which appreciation and full acceptance is less feasible or even not desirable, and this might matter for the implications that the experience of being tolerated has. Future research could examine whether tolerance is normative in a given context or not, and whether this distinction has a psychological and social impact on the person being tolerated [35].

In conclusion, a diverse, equal and inclusive setting depends on people’s willingness to allow others to have and express their own opinions, beliefs and personalities. It requires that people put up with things they disagree with, dislike or disapprove of, also with things that are antithetical and incompatible with their own convictions, commitments, and worldviews. In short, it requires tolerance as forbearance which is widely considered a critical component of managing diverse contexts [3]. However, there can be negative side effects that should be acknowledged and considered in finding balanced and productive ways for dealing with forms of diversity in organizations, institutions, and society more broadly.

## Supporting information

S1 File (DOCX)
