# Supplementary material for: The unintended consequences of tolerance: The experience and repercussions of being tolerated for minority group members
Source: PLoS One. 2023 Mar 22;18(3):e0282073. doi: 10.1371/journal.pone.0282073 (PMC10032513; doi:10.1371/journal.pone.0282073)
Supplement: S1 File — (DOCX) [file pone.0282073.s001.docx]

The Unintended Consequences of Tolerance: The experience and repercussions of being tolerated

**Supplemental online materials**

**Measures and Materials**

*Study 1*

- DEMOGRAPHICS
- What is your gender? (Female, Male, I prefer not to answer this question, Other)
- What is your age? (Under 12 years old, 12-17 years old, 18-25 years old, 26-35 years old, 36-45 years old, 46-55 years old, 56-65 years old, 66-74 years old, 75 years or older, I prefer not to answer this question.)
- Years employed? (Unemployed, Student, 0 years, 1-3 years, 4-6 years, 7-10 years, 11-15 years, 16-25 years, 26+ years, I prefer not to answer this question)
- PERSON VS. TASK ORIENTATION MEASURE
- Please indicate how often you would do the following if you were a team leader: (I would push team members for more effort., I would require team members to always use standard procedures that I decide upon., I would personally settle conflicts when they emerged in the team., I would decide alone which tasks team members should fulfill., I would allow members complete freedom in their work., I would refuse to explain my actions., I would keep the work moving at a rapid pace., I would persuade others that my ideas are to their advantage.)
- TRUST GAME
- The two of you are working in a manufacturing company that makes aircraft parts. You are both put on a project to provide a major order of aircraft parts to a large airliner company. Over the course of the project you each take a few shortcuts in your paperwork. All of a sudden, right before delivery, the project falls through and your supervisor wants to interview both of you about how the project went to see if she can discover any problems in your performance. You two are not able to talk to each other but you know that if neither of you says anything about the paperwork shortcuts, then nothing will happen to you. If both of you tell about the shortcuts, then both of you will see your pay scale reduced. However, if one of you but not the other tells the supervisor about the shortcuts, then the one who tells will keep their job and pay, and the other will be fired. Without being able to talk to your colleague, what would you do? Please indicate the response that best represents your opinion. (Tell the supervisor about the paperwork shortcut, Do not tell the supervisor about the paperwork shortcut)
- Both of you are working in an advertising company and together you put together a successful advertising campaign that leads a major clothing manufacturer to become a long-term company client. Your supervisor is thrilled and invites both you into his office separately to talk about your process, and you know that there may be raises, bonuses, or even promotions emerging from that meeting. However, you are not sure how you should explain who did the more impressive work. You are not able to talk to your colleague to know what they will say. If you both say that the work was equally performed, then both of you will receive a promotion. If both of you say that you did most of the work, then neither will receive a promotion. And if one of you says they did more of the work and the other says it was equal, the one who says they did more will receive the promotion. Without being able to talk to your colleague, what would you do? Please indicate the response that best represents your opinion. (Say that both of you contributed equally, Say that you did most of the work)
- Both of you have been assigned with the task of managing a taskforce of 15 workers to prepare a complex computer program. One of the members of the team is your best friend, and he has been going through a very difficult time and has not been fully completing his tasks. Your supervisor has called the managers to come to her office to discuss the progress of the project. You would like to protect your friend, since you know that losing his job would be the worst thing for him at this point. You don’t have a chance to speak to your fellow manager before you both go into the meetings. You know that if neither of you mentions the slow progress of your friend, his job will be fine. If both of you point out that he has been slow, the supervisor will probably get worried and call your friend in, and while he won’t be fired, he will have his position and pay reduced. However, if only one of you mention that he has been slow, the supervisor will just ask you to fire him. Without the chance to speak to your fellow manager, what would you do? Please indicate the response that best represents your opinion. (Tell the supervisor about your friend’s slow progress, Not tell the supervisor about your friend’s slow progress)
- EMOTIONS
- Please indicate how much you felt the following emotion while completing the team-building activity. (Upset, Motivated, Nervous, Sad, Inspired, Happy)
- PERSONAL TEAMWORK BELIEFS
- I believe I can succeed in a online teamwork environment., I am excited to work in online work teams in the future., I am confident in my abilities to be a good team member.
- FUTURE EXPECTATIONS
- You and the other members of your work-team are assigned to a group project that will require creativity in how to fulfill the task and co-ordination to combine a number of different smaller components of the larger project. Now imagine that you, as a people-oriented team member, want to suggest an approach to the group project that focuses on the social needs of the group members. (How much do you think your team members would listen to your suggestion with an open mind?, How much do you think your team members would value your suggestion?, How much do you think that your team members would incorporate your suggestion into the project?, How much do you think that your team members would ask you your suggestions in future projects?)
- WELL-BEING
- During the activities, I had the feeling that I belonged to the team., I did not feel accepted by the other team members., I felt like an outsider during the teamwork activities., I felt in control over the teamwork activities., I had the feeling that the other members of the team decided everything., Being part of this team made me feel insecure., I was concerned about what the other team members thought about me during the teamwork activities., I had the feeling that the other team members did not like me., I believed that my contribution to the teamwork activities did not matter., I would like to work with this team again., Our team does not work well together.
- MANIPULATION CHECK
- (I felt like my teammates valued me and included me., I felt like my teammates did not value me and left me out., I felt like my teammates did not value me, but put up with me.)
- OPEN-ENDED RAISING VOICE
- We understand that you were randomly assigned to a team and did not get to choose your fellow team members. Please let us know here what that experience was like for you or if you had any problems with any of your group members as we work to improve this research.
- ADDITIONAL DEMOGRAPHICS
- What is your race? Mark one or more races to indicate what you consider yourself to be. American Indian or Alaska Native (a person having origins in any of the original peoples of the Americas through tribal affiliation or community attachment)", Asian (a person having origins in any of the original peoples of the Far East, Southeast Asia, or the Indian Subcontinent, including, for example, Cambodia, China, India, Japan, Korea, Malaysia, Pakistan, the Philippine Islands, Thailand, and Vietnam)", Black or African American (a person having origins in any of the black racial groups of Africa)",Native Hawaiian or Other Pacific Islander (a person having origins in any of the original peoples of Hawaii, Guam, Samoa, or other Pacific Islands)",White (a person having origins in any of the original peoples of Europe)",Latino/Latina (a person having origins in Latin America, Central America, or Mexico)"I prefer not to answer this question")
- CYBERBALL ATTENTION CHECK
- During the ball-passing exercise, how much did your fellow team-members include you? (Never, Rarely, Occasionally, Equally, Frequently, I don't remember)

*Study 2*

- EMOTION
- (Lonely, Unwanted, At ease, Dependent, Confident, Happy, Looked down upon, Supported)
- RAISING VOICE
- During the teamwork exercises, were your teammates uncooperative or otherwise a problem?" ("Yes, all of them","Yes, some of them","Yes, but only one of them""No, none of them")
- Based on your experiences, would you recommend that all or some of your teammates be excluded from participating in future teams? ("Definitely yes","Maybe yes","Maybe not""Definitely not")
- FUTURE WITHDRAWAL
- (how likely would you be to put your suggestion forward on your own initiative?, how likely you be to withdraw and not engage with your teammates?, how likely would you be to discuss your differences of opinion with your teammates?, how likely would you be to ignore your idea and focus on something else instead?)
- WELL-BEING
- (I had the feeling that I belonged to the team., I felt left out by the other team members., I felt lonely during the teamwork activities., I felt in control over the teamwork activities., I had the feeling that the other members of the team decided everything., I felt that I had less control over the situation than the others., I felt that the other team members really valued me., I had the feeling that the other team members did not really like me., I felt disrespected during the teamwork activities., I was not worried about what to expect from the other team members., I felt uncertain about fitting in with the team., I felt uncertain about how the other team members would behave towards me.
- MANIPULATION CHECK
- I felt like my teammates fully included me and fully valued me., I felt like my teammates completely left me out and did not value me at all., I felt like my teammates merely put up with me, and did not fully value me.

*Study 3*

- DEMOGRAPHICS
- Were your parents born in the Netherlands? (Both, One, Neither, Prefer not to answer)

*Study 4*

- Public review: Online Workgroups: Teamwork and Leadership
  - Did the training study help you connect to your workgroup?
  - Did you benefit from the workgroup experience?
  - Overall, how did you feel about the training study?
  - Describe Your Experience
  - Would you recommend this training study?
    - Yes!
    - No
- Web-address: <https://quillup.com/rate-review/>

**Sample comics used in the studies:**


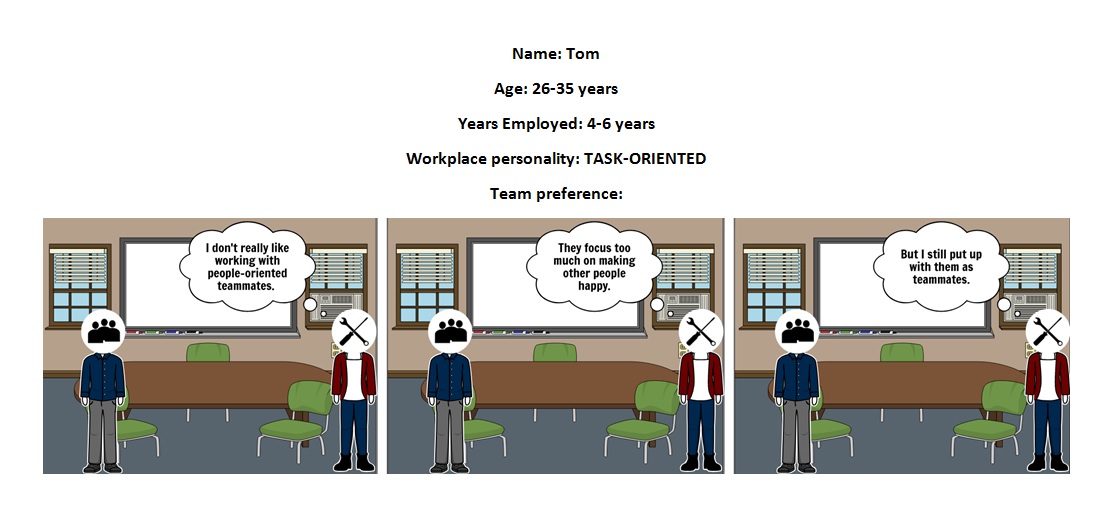


This image depicts the information players were presented with about one of their teammates. In this case, the teammate, Tom, is being presented to a participant in the Tolerance Condition.


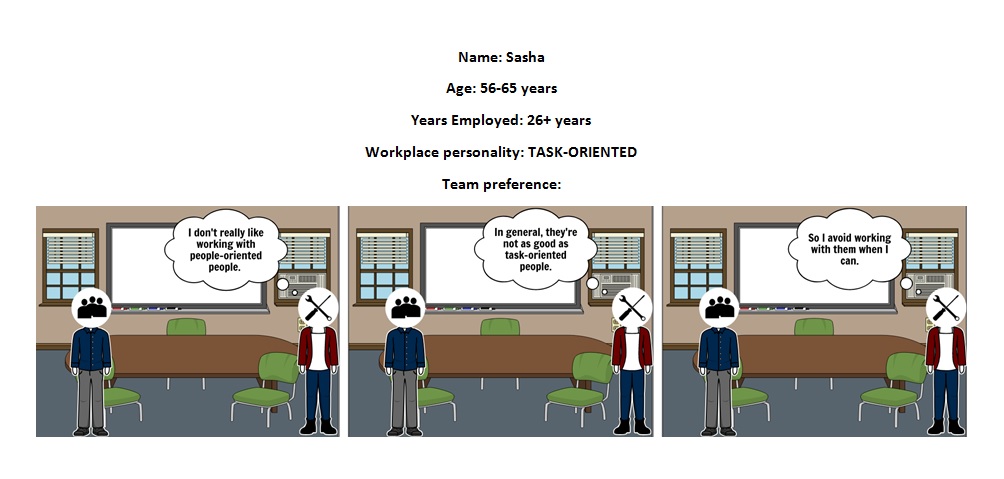


In this example, the player (who is in the rejection condition) is being introduced to Sasha.
